# LXNet: A lightweight CNN for lung disease classification from Chest X-ray with XAI-based interpretability

**DOI:** 10.1371/journal.pone.0351762

**Published:** 2026-06-17

**Authors:** Juiria Humayan, Md. Najmus Sakib Nahid, Amir Sohel, Md Alamgir Kabir, Md Shakhawat Hossain, Zahid Ullah, Mona Jamjoom

**Affiliations:** 1 Department of Computer Science and Engineering, Daffodil International University, Dhaka, Bangladesh; 2 School of Informatics, Kochi University of Technology, Kami, Kochi, Japan; 3 Information Systems Department, College of Computer and Information Sciences, Imam Mohammad Ibn Saud Islamic University (IMSIU), Riyadh, Saudi Arabia; 4 Department of Computer Sciences, College of Computer and Information Sciences, Princess Nourah bint Abdulrahman University, Riyadh, Saudi Arabia; Najran University College of Computer Science and Information Systems, SAUDI ARABIA

## Abstract

The diagnosis of lung diseases such as pneumonia and tuberculosis remains a major global health challenge, especially in resource-limited regions. Artificial Intelligence (AI) has shown strong potential in analyzing Chest X-Rays (CXR) for accurate and timely diagnosis, but most existing models are computationally heavy and lack interpretability, limiting their practical application. In this study, we present LXNet, a lightweight and explainable Convolutional Neural Network (CNN) for nine-class lung disease classification (*Normal*, *Pneumonia*, *Higher Density*, *Lower Density*, *Obstructive Pulmonary Diseases*, *Degenerative Infectious Diseases*, *Encapsulated Lesions*, *Mediastinal Changes* and *Chest Changes*). The model was evaluated on a diverse CXR dataset containing 6,743 images collected from a private imaging center (GRS Imagem, Brazil), enabling comprehensive multiclass assessment. LXNet contains only 0.35 million parameters and employs a no-pooling final block to preserve subtle diagnostic features while maintaining very low computational cost. Robustness was enhanced through adaptive Contrast Limited Adaptive Histogram Equalization (CLAHE), grayscale normalization and stratified class balancing. LXNet was benchmarked against pretrained CNNs (DenseNet201, ResNet50V2 and InceptionV3) under identical settings. Explainable AI (Grad-CAM, Score-CAM and LIME) provided meaningful visualizations. LXNet achieved 96.1% accuracy in 5-fold cross validation, outperforming the baselines (DenseNet201: 90.3%, InceptionV3: 88.9%) by 1–8%, with only 308 seconds of training on standard hardware. Statistical significance was confirmed using Wilcoxon signed-rank tests (*p* = 0.03125). These results demonstrate LXNet’s promising performance and interpretability; however, reduced external performance indicates limited generalizability and its clinical applicability requires further validation.

## Introduction

Lung diseases remain a primary global health concern, contributing significantly to morbidity and mortality worldwide. According to the World Health Organization (WHO), tuberculosis alone accounts for over 1.3 million deaths annually [1], while respiratory infections such as pneumonia and COVID-19 continue to place substantial burdens on healthcare systems. Especially when we have a shortage of healthcare professionals worldwide. In developing countries like Bangladesh, the situation is further exacerbated by widespread tuberculosis and repeated outbreaks of viral pneumonia and COVID-19, which put additional pressure on already limited healthcare resources [2]. On top of that, in Bangladesh, the doctors-to-patients ratio is 1:1901, indicating poor access to and quality of healthcare facilities [3]. Automated diagnosis tools can significantly improve healthcare access and quality regardless of the region. However, despite the global disease burden and the shortage of trained radiologists, particularly in low-resource countries, existing AI-based diagnostic models are computationally complex and lack the transparency required for routine clinical adoption. This study directly addresses these limitations by proposing a lightweight, interpretable and clinically deployable model capable of accurately detecting multiple lung diseases from Chest X-Rays (CXRs).

Recent advances in deep learning have enabled the development of automated systems capable of analyzing CXRs with high accuracy, offering valuable assistance to radiologists and improving diagnostic consistency [4]. Deep learning models, particularly Convolutional Neural Networks (CNNs) and hybrid architectures, have become the dominant approach for medical image analysis. These AI models automatically learn discriminative features from imaging data, eliminating the need for manual feature extraction and significantly reducing labor and dependency on healthcare professionals. Lack of trust in the AI is another critical issue for the practical use of AI-driven diagnosis tools. Approximately, 60% of American adults are not comfortable using AI for their healthcare [5]. Studies have shown that integrating explainable AI (XAI) techniques such as Gradient-Weighted Class Activation Mapping (Grad-CAM), Score-Weighted Class Activation Mapping (Score-CAM) and Local Interpretable Model-Agnostic Explanations (LIME) into CNN frameworks enhances model transparency and fosters clinical trust [6]. Moreover, the design of lightweight, domain-specific architectures tailored for lung disease classification has become a key research focus, balancing accuracy, computational efficiency and real-world applicability. Such innovations promise scalable, real-time diagnostic solutions for diverse healthcare environments [7,8]. Recent advances in 2025 include reciprocal attention mechanisms for rapid heatmap generation and lightweight architectures with privacy-preserving federated training, further improving diagnostic performance and interpretability [9,10]. However, many of these methods still produce coarse, post-hoc visual explanations that do not fully align with radiological reasoning, limiting their practical utility in clinical workflows. Unlike existing methods, our LXNet framework generates class-specific, fine-grained saliency maps that reliably highlight radiologically meaningful patterns in the image, such as consolidations, opacities and cavitary. This enables clinicians to visually validate the model’s predictions in real time.

In recent years, deep learning techniques have shown remarkable progress in automating CXR-based disease detection, enabling rapid and accurate clinical decision support. Numerous CNNs and transfer learning approaches, such as ResNet, DenseNet, Inception, and MobileNet variants, have been employed for lung disease classification [4,7–10]. Recent research has shifted toward lightweight architectures [10,11] hybrid deep learning frameworks [7,12] and XAI-based models [7,8,12] to enhance interpretability and facilitate clinical deployment. The latest studies have introduced reciprocal saliency mapping and federated attention mechanisms to further improve transparency and scalability in real-world applications [9,10]. Nevertheless, only a limited number of these studies rigorously evaluate their models on diverse, real-world datasets, particularly those reflecting resource-constrained environments like Bangladesh, leaving a gap in assessing true clinical deployability.

Despite these advances, key challenges persist, including class imbalance [8,13], high computational demand [14,15], limited interpretability [16] and inconsistent evaluation protocols across datasets [1,2]. Research from 2020 to 2025 has increasingly focused on not only boosting predictive accuracy but also lowering computational requirements and improving the scalability, generalization, and transparency of lung disease classification models [4,7,9,10,12,15,17–20]. Recent lightweight CNNs with rapid CAM-style explanations and privacy-preserving federated training have demonstrated promising deployment potential in clinical and CT-based cohorts, underscoring the growing need for efficient, interpretable, and resource-aware diagnostic pipelines [9,10,18–20].

Although large pretrained CNNs achieve high predictive performance, their deployment in real-world medical environments is often limited by substantial computational requirements and poor interpretability. Existing lightweight architectures improve efficiency but frequently compromise accuracy and robustness. Explainability is another issue which limits their practical use. Therefore, there is a strong motivation to design a lightweight, accurate, and explainable CNN framework for multi-class lung disease classification that can perform reliably in resource-constrained healthcare settings. This motivates the development of a compact yet robust model capable of preserving diagnostic accuracy under computational limitations, minimizing critical errors such as false negatives, and maintaining generalizability across diverse datasets and clinical environments [9,10,18–20]. In summary, the existing literature lacks a model that is simultaneously lightweight, highly accurate, interpretable and validated on realistic, diverse datasets, representing the precise gap this study aims to fill.

Despite notable progress, several key technical gaps remain in lung disease classification research. Firstly, many deep learning models are computationally demanding, containing millions of parameters that hinder real-world deployment in resource-limited healthcare environments [4,14,17]. Large architectures such as ResNet [11] and DenseNet [21] deliver strong performance but require significant memory and processing power, making them unsuitable for time-critical or low-resource settings. Secondly, although these models achieve high predictive accuracy, their limited interpretability continues to restrict practical use. Studies by Shah et al. [8], Bhandari et al. [22], and Ifty et al. [12] emphasize the importance of XAI integration within CNN frameworks. However, most existing approaches remain generic and are not sufficiently adapted to the complexities of medical image interpretation, thereby limiting reliability. Thirdly, many frameworks rely on large annotated datasets and complex preprocessing pipelines, which constrain scalability and usability, particularly in developing healthcare systems where data curation and computational infrastructure are limited [23–25]. Fourthly, ensuring robust generalization across datasets, imaging devices and noise conditions remains a critical challenge. As noted by Alshmrani et al. [15] and Hong et al. [26], models trained on specific datasets often fail to maintain consistent performance across diverse clinical cohorts and imaging environments. Fifthly, there remains a lack of lightweight, domain-specific architectures optimized for efficient, real-time lung disease diagnosis. Existing lightweight CNNs often trade off accuracy or robustness for efficiency, limiting their suitability for clinical workflows [7,8,27]. Finally, while recent advances such as compact CNNs, rapid XAI mechanisms and federated learning have shown promise toward deployable and privacy-preserving solutions [9,10], significant issues persist. These include limited generalizability across cohorts, insufficient clinical validation and difficulty minimizing false negatives without increasing model complexity. Moreover, hybrid attention-based models, though accurate, often elevate computational cost and reduce interpretability, which can hinder routine clinical adoption [19,20]. Therefore, the need for an accurate, interpretable and lightweight AI model for lung disease diagnosis from CXR images remains critical to support practical and deployable diagnostic solutions. **This study makes the following key contributions:**

- We present LXNet, a compact and task-optimized CNN framework for multi-class lung disease diagnosis from CXRs. Rather than introducing new architectural components, LXNet carefully integrates established CNN design choices into a lightweight configuration with only 0.35 million trainable parameters. The use of a no-pooling final block is an engineering-driven design choice to preserve fine-grained diagnostic features while maintaining computational efficiency, making the model suitable for resource-limited environments.
- We performed a comprehensive and systematic evaluation of LXNet to assess its practical use in hospitals. The model is benchmarked against widely used pretrained CNNs (DenseNet201, ResNet50V2 and InceptionV3) under identical experimental conditions. In addition, five-fold cross-validation, time complexity analysis, statistical significance testing and interpretability assessments using Grad-CAM, Score-CAM and LIME are conducted to examine performance robustness, reliability and transparency.

## Related work

CXRs remain the most widely used imaging modality for diagnosing lung diseases due to their affordability, accessibility, and diagnostic value [1]. Building on this foundation, advances in deep learning have enabled automated frameworks that substantially improve classification performance, progressing from early SVM, KNN and decision tree baselines toward CNNs and transfer learning using architectures such as ResNet [11], DenseNet [21], and MobileNet [14]. More recent research has emphasized custom lightweight CNNs [17,22], hybrid deep learning strategies [28] and XAI approaches [12] to mitigate the challenges of computational cost, class imbalance and limited interpretability.

Several studies have reported high accuracy but also identified constraints on scalability and generalization. For instance, Mohan et al. [25] achieved 93–97% accuracy across two public datasets using three CNN variants but observed performance variations due to differences in image acquisition conditions. Alshmrani et al. [15] developed a deep CNN for detecting tuberculosis and COVID-19, improving feature representation but increasing computational overhead. Similarly, Hao et al. [26] applied EfficientNet-B7 with noisy student training, achieving 96.1% accuracy while emphasizing the importance of balanced datasets. In contrast, Metwally et al. [13] compared EfficientNetB0, Xception, and NasNetLarge for four-class classification, identifying EfficientNetB0 as the most cost-effective despite a slight accuracy trade-off.

To enhance deployability, subsequent studies have focused on lightweight and interpretable designs. Shamrat et al. [17] introduced MobileLungNetV2, a customized MobileNetV2 variant that surpassed 96% accuracy but required significant training time. Kim et al. [29] employed EfficientNetV2-M for pneumonia and pneumothorax classification, achieving competitive accuracy but offering limited interpretability. Likewise, Bhandari et al. [22] developed a compact CNN for COVID-19, pneumonia, and tuberculosis, emphasizing explainability to gain the trust of healthcare professionals and patients and achieve better clinical deployability. Al-Sheikh et al. [27] combined custom CNNs with ensembling across X-ray and CT modalities, achieving nearly 99% accuracy, though they were still challenged by inconsistent image quality. Ifty et al. [12] integrated Xception with XAI (Grad-CAM and LIME), achieving 96.21% accuracy and demonstrating that visual explanations can strengthen clinician confidence.

Hybrid and multi-label learning have also attracted attention for multi-condition diagnosis. Farhan and Yang [28] presented a hybrid ResNet50–SVM/AdaBoost framework that achieved 98.99% accuracy but lacked real-time capability. Irtaza et al. [30] applied multiple backbones to the NIH CXR dataset for multi-label classification, improving generalization through Generative Adversarial Network (GAN)- based augmentation, but at the cost of high computational overhead. From a deployability standpoint, Aldamani et al. [31] proposed LungVision, an EfficientNetB2 model with quantization, which attained a 98.58% F1-score and edge-device compatibility, though interpretability remained limited. Comparative analyses further highlight these trade-offs. Hamal et al. [18] compared traditional shallow machine learning with deep learning models for COVID-19 and pneumonia detection, finding that CNNs were superior at feature learning but less generalizable across datasets. Tian et al. [20] used DenseNet-161 with hybrid attention and clinical metadata for MRI-based tumor classification (92.44% accuracy; AUC of 0.971), highlighting similar computational and interpretability challenges. Bondugula et al. [19] proposed a transfer learning model that prioritizes reducing false negatives in infectious disease detection, achieving high accuracy but at the cost of increased architectural complexity, which is unsuitable for low-resource environments.

More recent directions have prioritized compactness, transparency and privacy for clinical readiness. Choi and Yang [9] introduced a lightweight CNN, ReciproCAM, achieving approximately 99% accuracy and providing rapid heatmap explanations for practical use. Saha et al. [10] developed Lung-AttNet, integrating global attention and federated learning to achieve 91% accuracy while preserving privacy across multiple sites. Nonetheless, these approaches still face challenges in maintaining accuracy under distributed conditions and ensuring interpretability suitable for clinical workflows. Similarly, Krishnamoorthy et al. [32] combined NASNet architectures with LIME for superpixel-level XAI, advancing transparency but remaining constrained by generalization and deployment issues.

Despite these significant advances in deep learning and explainable frameworks for CXR-based lung disease diagnosis, several critical gaps remain: (1) high computational demands that hinder real-time use in resource-limited settings [15,17]; (2) poor handling of data imbalance and domain variation [26,30]; (3) inconsistent image quality and lack of robust interpretability integration [12,27]; and (4) limited validation of lightweight, XAI-based CNNs across diverse cohorts [9,10]. Addressing these open issues, this study proposes LXNet, a lightweight and explainable CNN architecture optimized for nine-class lung disease classification from CXRs, aiming for real-world applicability in resource-constrained healthcare centers.

## Materials and methodology

**Fig 1:**
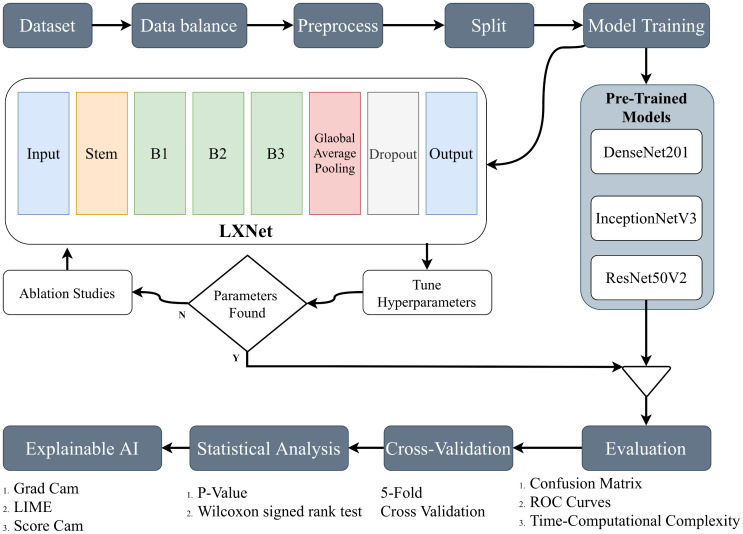
End-to-end experimental workflow illustrating stratified data splitting, preprocessing, uniform model training, evaluation and XAI analysis.

This study was systematically designed to enable a comprehensive and rigorous evaluation of the proposed LXNet architecture for multi-class lung disease classification using CXR images. Initially, the lung CXR dataset was balanced and preprocessed to ensure high-quality inputs suitable for model training. Subsequently, an extensive ablation study was conducted to identify the optimal hyperparameter combination, including activation function, learning rate, batch size and optimizer settings. **The optimized LXNet was then evaluated across multiple stages: (1) comparative analysis with established baseline CNN architectures, including ResNet50V2, DenseNet201 and InceptionV3; (2) detailed class-wise performance assessment on the lung disease dataset; and (3) five-fold cross-validation to evaluate robustness and generalizability**. Further experiments included benchmarking LXNet against recent lung disease classification methods and conducting a time complexity analysis to assess computational efficiency relative to pretrained baselines. To ensure the statistical validity of the results, statistical significance testing was performed. Finally, interpretability analyses using Grad-CAM, Score-CAM and LIME were applied to visualize and validate LXNet’s decision-making process. The complete experimental workflow, encompassing dataset balancing, preprocessing, stratified splits, model training, evaluation and explainability analysis, is illustrated in Fig 1.

### Ethical approval

The dataset employed in this study comprises anonymized human CXR images that are publicly available on Kaggle (https://www.kaggle.com/datasets/fernando2rad/x-ray-lung-diseases-images-9-classes). As all personal identifiers were removed and the data were fully de-identified, the university research ethics committee waived the requirement for Institutional Review Board (IRB) approval. This study does not involve direct interaction with human participants or access to identifiable private information, thereby adhering to established ethical standards for human subjects research.

### Dataset description

This study employs a widely used CXR lung disease dataset comprising 9 classes [23] and 6,743 X-ray images. The dataset includes *Normal* (1,340 images), *Pneumonia* (1,060 images), *Higher Density* (678 images), *Lower Density* (629 images), *Obstructive Pulmonary Diseases* (644 images), *Degenerative Infectious Diseases* (594 images), *Encapsulated Lesions* (658 images), *Mediastinal Changes* (596 images) and *Chest Changes* (544 images). This distribution is diverse, but the classes were not balanced. Therefore, we balanced the dataset, allowing the model to learn subtle variations across a broad spectrum of pulmonary conditions. The images were collected from a private imaging center (GRS Imagem) in Brazil. The *Higher Density* class includes radiological conditions such as pleural effusion, atelectatic consolidation, hydrothorax and empyema. The *Lower Density* class corresponds to conditions involving abnormal air accumulation, including pneumothorax, pneumomediastinum and pneumoperitoneum. *Obstructive Pulmonary Diseases* comprise emphysema, bronchopneumonia, bronchiectasis and pulmonary embolism. *Degenerative Infectious Diseases* include chronic progressive conditions such as tuberculosis, sarcoidosis, proteinosis and pulmonary fibrosis. *Encapsulated Lesions* cover well-defined localized abnormalities, including abscesses, nodules, cysts, tumor masses and metastases. *Mediastinal Changes* represent abnormalities such as pericarditis, arteriovenous malformations and lymph node enlargement. *Chest Changes* include structural abnormalities caused by conditions such as atelectasis, congenital malformations, agenesis and hypoplasia. It is important to note that the utilized dataset does not provide comprehensive patient-level information, including demographic and clinical details such as age, sex, ethnicity or medical background. In addition, the absence of patient identifiers restricts the ability to verify patient-wise independence during data partitioning. These limitations constrain the assessment of dataset diversity, representativeness and potential bias and should be considered when interpreting the generalizability of the proposed model. However, because the primary objective of this study is to evaluate the multiclass classification performance of the proposed model across diverse radiological categories, the dataset remains appropriate and technically suitable for benchmarking classification accuracy and comparative performance.

Data augmentation was applied to address class imbalance and improve model generalization while strictly preserving the diagnostic integrity of CXR images. All augmentation parameters were carefully selected to reflect realistic variations encountered during image acquisition and patient positioning, thereby ensuring medical plausibility. The augmentations included rotations (−5° to +5°) to account for minor patient misalignment during imaging, zoom-in scaling (1.05× to 1.15×) to simulate variations in source-to-detector distance and horizontal translations up to 5% of the image size to reflect slight shifts in patient centering. Horizontal flipping was selectively employed, as left-right anatomical symmetry in CXRs is commonly used in the literature and does not compromise diagnostic validity for the studied conditions. Vertical flipping was intentionally excluded because it would invert anatomical orientation and compromise diagnostic relevance. All transformations were performed using bilinear interpolation to maintain image quality and avoid artificial artifacts. These augmentation strategies enhance model robustness by enabling the network to learn stable and invariant features under minor variations in image orientation, patient positioning, scaling and centering that commonly occur during real-world CXR acquisition.

After augmentation, 1,000 images were randomly selected per class, ensuring that 50% (500 images) were original and the remaining 50% were augmented versions derived exclusively from the training pool. This resulted in a balanced dataset of 9,000 images (1,000 per class). For hold-out validation, 80% (7,200 images) were used for training, 10% (900 images) for validation, and 10% (900 images) for testing. We ensured that no test images or their augmented variants were present in the training or validation sets.

**Fig 2:**
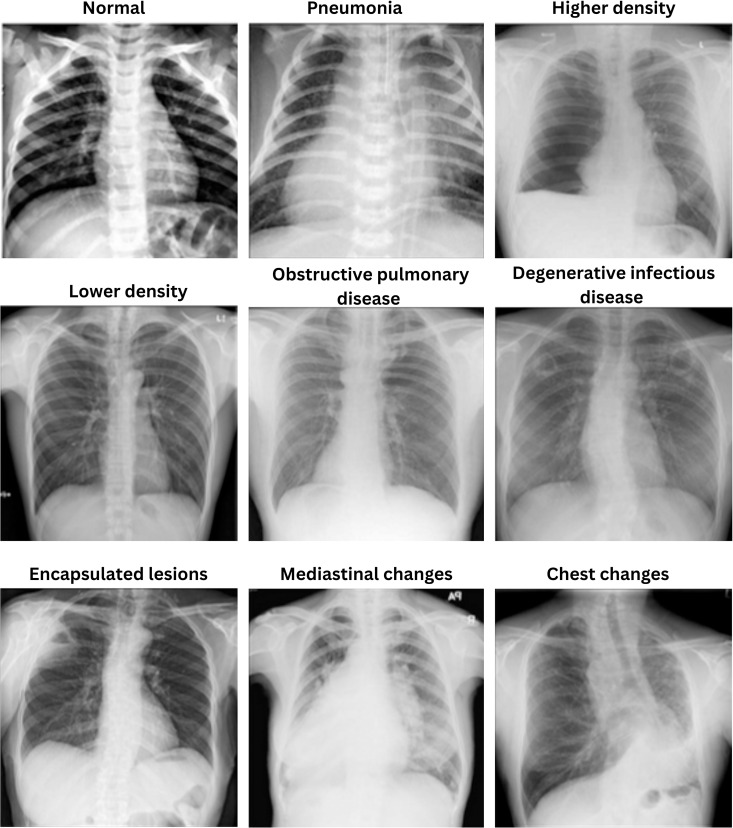
Sample CXR image of nine classes used in this study.

For five-fold cross-validation, the complete set of 9,000 images was stratified into five groups of 1,800 images each, preserving class distributions across folds, which is critical for reliable multiclass medical image evaluation. The hold-out dataset was utilized for ablation studies, hyperparameter optimization, class-wise performance analysis, computational complexity evaluation, comparison with recent lung image classification methods and interpretability analyses. In contrast, the cross-validation dataset was employed exclusively for 5-fold performance estimation and statistical significance testing. Sample images from the dataset are illustrated in Fig 2.

### Reproducibility

The hold-out validation experiments were conducted using a fixed random seed (seed = 42) for Python, NumPy, and TensorFlow to ensure reproducibility across all models, including the proposed LXNet. The cross validation experiments were conducted using three random seeds (42, 123 and 999). Experiments were implemented in Python 3.10 using TensorFlow/Keras 2.x on a cloud-based environment (Kaggle) utilizing dual NVIDIA Tesla T4 GPUs.

### Pre-processing

The preprocessing pipeline was designed to standardize and enhance the quality of input CXR images before model training. All images were first resized to a fixed dimension to ensure consistent input resolution, followed by contrast enhancement using Contrast Limited Adaptive Histogram Equalization (CLAHE) to emphasize relevant local structures while minimizing noise amplification and artifact generation. Finally, pixel intensities were normalized to a standard scale to stabilize optimization and improve training efficiency. Together, these preprocessing steps reduce variability caused by differences in image acquisition, illumination and contrast, thereby improving the robustness and consistency of feature extraction across heterogeneous CXR images.

### Resizing

The CNN models require fixed-size inputs. Since the original CXR images varied in resolution, all images were resized to 224 × 224 pixels to match the input size expected by the pretrained networks. Resizing was performed using bilinear interpolation, where the intensity of each new pixel *I*(*x*, *y*) is computed as a weighted average of the four nearest pixels in the original image, as expressed in Eq. 1:


$$\begin{array}{c} I(x,y)=\sum_{i=1}^{2}\sum_{j=1}^{2}w_{ij}\;I(x_{i},y_{j}) \end{array}$$ (1)

where $w_{ij}$ are the interpolation weights determined by the relative distances between the new pixel and its neighboring pixels. This approach preserves image smoothness and minimizes distortion.

### Contrast Enhancement using CLAHE

CLAHE enhances local contrast by dividing an image into small contextual regions (tiles) and independently applying histogram equalization to each. The *clip limit* parameter constrains the amplification of noise, while the *tile grid size* determines the number of regions processed. In this study, CLAHE was applied to improve the visibility of subtle features in CXRs.

Unlike conventional CLAHE implementations that use fixed parameter settings, our method employs a grid search optimization to systematically explore combinations of *clip limit* and *tile grid size*. For each image, the configuration yielding the highest local contrast enhancement was automatically selected, ensuring improved feature visibility without introducing excessive noise. The CLAHE transformation is mathematically expressed in Eq. 2:


$$\begin{array}{c} s=(L-1)\times CDF(r) \end{array}$$ (2)

where *L* denotes the number of gray levels, and *CDF*(*r*) represents the clipped and redistributed cumulative distribution function.

**Fig 3:**
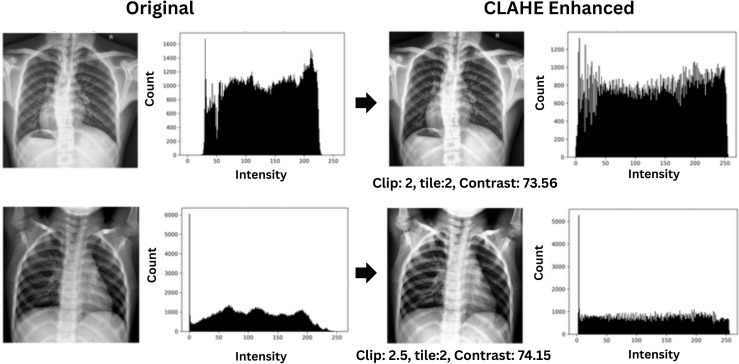
Adaptive CLAHE based enhancement of CXR images.

In Fig 3, shows the impact of CLAHE on the CXR images. The grid search-based CLAHE produced a noticeable improvement in local contrast, particularly in regions exhibiting subtle diagnostic details.

### Normalization

CNNs typically perform better when input intensities are scaled to a consistent range. The original pixel values were normalized to the interval [0, 1] by dividing each pixel value *p* by 255, shown in Eq. 3:


$$\begin{array}{c} p^{\prime }=\frac{p}{255} \end{array}$$ (3)

This normalization ensures numerical stability during training and accelerates model convergence.

### Architecture of the Proposed LXNet

**Fig 4:**
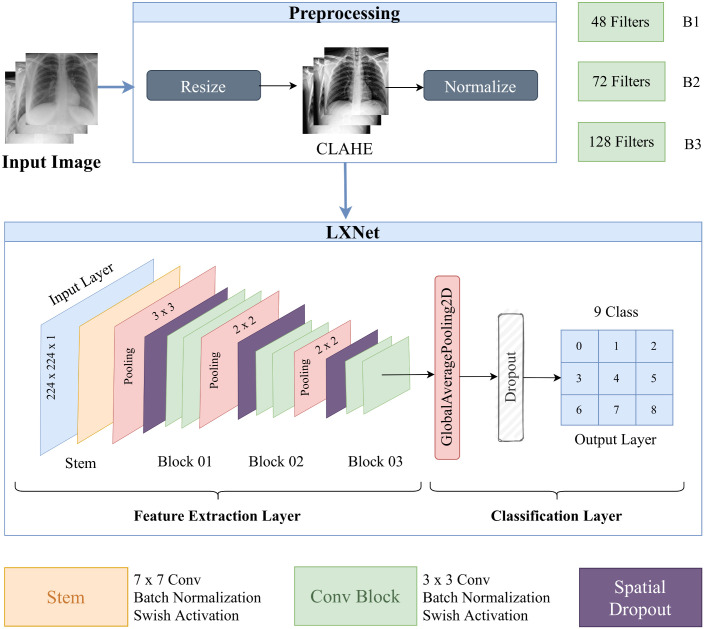
LXNet pipeline and lightweight architecture for nine‑class chest X‑ray classification.

Fig 4 illustrates the overall LXNet architecture and its major components: (1) preprocessing and data ingestion, (2) the LXNet feature-extraction backbone and (3) the classification head. In the left-most block, the raw CXR image ($I\in {\mathbb{R}}^{H\times W\times 1}$) is fed into an adaptive preprocessing stage composed of resizing, CLAHE-based contrast enhancement (with parameters selected via grid search per image) and intensity normalization. Then, the preprocessed images are fed into the LXNet backbone, which begins with a 7 × 7 stem convolution (32 filters) to capture low-level edges and textures. This is followed by three hierarchical convolutional blocks:

- **Conv Block 1:** Two 3 × 3 convolution layers (48 filters), each followed by Batch Normalization and Swish activation, then Max Pooling and SpatialDropout2D to reduce co-adaptation.
- **Conv Block 2:** Two 3 × 3 convolution layers (72 filters), with the same BN + Swish + Max Pool + Spatial Dropout pattern to capture mid-level shapes and contours.
- **Conv Block 3:** Two 3 × 3 convolution layers (128 filters) that prioritize high-level semantic features (no pooling or dropout here to retain spatial detail needed to discriminate subtle pathologies).

After these blocks, a Global Average Pooling (GAP) layer compresses feature maps into a compact 128-dimensional vector, followed by a Dropout layer (rate = 0.3) and a final Dense Softmax classifier that outputs probabilities across nine disease classes. Fig 4 also highlights the small model footprint (∼0.35M trainable parameters) and the evaluation branch, where LXNet is benchmarked against DenseNet201, ResNet50V2 and InceptionV3 using accuracy, precision, recall, F1-score, confusion matrix, training time, 5-fold cross-validation and statistical significance testing. Finally, the XAI analysis (Grad-CAM, Score-CAM, LIME) produces heatmaps and superpixel-based visual explanations for qualitative analysis and interpretation.

### Stem convolution layer

The feature extraction process begins with a 7 × 7 convolutional stem layer with 32 filters, responsible for capturing fundamental edge- and texture-level features as expressed in Eq. 4:


$$\begin{array}{c} X_{1}=f(W^{\left(1\right)}*X_{0}+b^{\left(1\right)}) \end{array}$$ (4)

where $W^{\left(1\right)}\in {\mathbb{R}}^{7\times 7\times 1\times 32}$, and f(·) denotes the Swish activation function, defined in Eq. 5:


$$\begin{array}{c} f(x)=x\cdot \sigma (x),\;\sigma (x)=\frac{1}{1+e^{-x}} \end{array}$$ (5)

Here, *f*(*x*) represents the Swish activation, *x* is the input value, and σ(x) is the sigmoid function. The Swish activation provides a smooth, non-monotonic transformation that enhances gradient flow and improves feature representation during training.

### Convolutional Block 1 (48 filters)

The first block refines low-level feature maps through two consecutive 3 × 3 convolutions with Batch Normalization and Swish activations as expressed in Eq. 6:


$$\begin{array}{c} X_{2}^{\left(i\right)}=f(BN(W^{\left(i\right)}*X_{2}^{\left(i-1\right)}+b^{\left(i\right)})),\;i=1,2 \end{array}$$ (6)

where *X*^(*i*)^ is the output feature map of the *i*-th convolutional layer in the block, *W*^(*i*)^ and *b*^(*i*)^ are the convolution kernel and bias, $X^{\left(i-1\right)}$ is the input to the *i*-th convolution, * denotes the convolution operation, BN(·) represents batch normalization, and f(·) is the Swish activation function defined in Eq. 5.

A max pooling operation reduces spatial dimensions while preserving dominant activations as expressed in Eq. 7:


$$\begin{array}{c} X_{2}^{\text{pool}}(p,q)=\max_{(m,n)\in R_{p,q}}X_{2}^{\left(2\right)}(m,n) \end{array}$$ (7)

where $X_{2}^{\text{pool}}(p,q)$ is the pooled output value at spatial position (*p*, *q*), $X_{2}^{\left(2\right)}(m,n)$ represents the input feature map before pooling, and $R_{p,q}$ denotes the set of spatial indices (e.g., a 2 × 2 window) associated with each pooled output location.

Subsequently, SpatialDropout2D is applied to deactivate entire feature maps, mitigating co-adaptation as expressed in Eq. 8:


$$\begin{array}{c} {\tilde{X}}_{2}=X_{2}^{\text{pool}}⊙Z,\;Z\sim Bernoulli(p=0.7) \end{array}$$ (8)

where ${\tilde{X}}_{2}$ is the resulting feature map after applying spatial dropout, $X_{2}^{\text{pool}}$ denotes the pooled feature map, ⊙ indicates element-wise multiplication, and *Z* is a binary mask generated independently for each channel by a Bernoulli distribution with keep probability 0.7.

### Convolutional Block 2 (72 filters)

The second block expands feature diversity and captures mid-level anatomical structures. Its formulation mirrors Block 1, given in Eq. 9:


$$\begin{array}{c} X_{3}^{\left(i\right)}=f(BN(W^{\left(i\right)}*X_{3}^{\left(i-1\right)}+b^{\left(i\right)})),\;i=1,2 \end{array}$$ (9)

where all symbols are as defined in Eq. 6, with an increased number of filters (72) to learn higher-level features in the second convolutional block.

### Convolutional Block 3 (128 filters)

The final convolutional block is designed for high-level semantic abstraction, enabling the network to capture disease-specific radiographic signatures (e.g., opacities, lesions, density variations), as given in Eq. 10:


$$\begin{array}{c} X_{4}^{\left(i\right)}=f(BN(W^{\left(i\right)}*X_{4}^{\left(i-1\right)}+b^{\left(i\right)})),\;i=1,2 \end{array}$$ (10)

where all symbols are as defined in Eq. 6, with an increased number of filters (128). This block does not include pooling or dropout to preserve fine spatial details in the feature maps.

### Global Average Pooling (GAP)

To reduce dimensionality while retaining class-discriminative features, a Global Average Pooling operation is performed as expressed in Eq. 11:


$$\begin{array}{c} g_{k}=\frac{1}{H\times W}\sum_{i=1}^{H}\sum_{j=1}^{W}X_{4}(i,j,k),\;g\in {\mathbb{R}}^{128} \end{array}$$ (11)

where $g_{k}$ denotes the global average pooled feature for channel *k*, *X*_4_(*i*, *j*, *k*) is the value at spatial location (*i*, *j*) in channel *k* of the final feature map, *H* and *W* are the height and width dimensions, and *g* is the resulting feature vector of dimension 128.

### Dropout Layer (rate = 0.3)

Dropout is applied to the pooled vector to further enhance generalization and reduce overfitting, expressed in Eq. 12:


$$\begin{array}{c} \tilde{g}=g⊙Z,\;Z\sim \text{Bernoulli}(p=0.7) \end{array}$$ (12)

where $\tilde{g}$ represents the feature vector after applying dropout, *g* is the globally pooled feature vector from Equation (9), ⊙ indicates element-wise multiplication, and *Z* is a binary mask vector with each element sampled from a Bernoulli distribution with probability 0.7.

### Softmax Classifier

Finally, a fully connected dense layer with softmax activation produces probabilistic outputs for the nine disease categories, expressed in Eq. 13:


$$\begin{array}{c} {\hat{y}}_{c}=\frac{\exp(w_{c}^{⊤}\tilde{g}+b_{c})}{\sum_{j=1}^{9}\exp(w_{j}^{⊤}\tilde{g}+b_{j})},\;c=1,\ldots ,9 \end{array}$$ (13)

where ${\hat{y}}_{c}$ denotes the predicted probability for class *c*, $w_{c}$ and $b_{c}$ are the weights and bias corresponding to class *c* in the final fully connected (dense) layer, $\tilde{g}$ is the feature vector after dropout, and the denominator sums the exponentiated scores over all nine classes to ensure the outputs form a valid probability distribution.

### Baseline architectures for comparison

To evaluate LXNet fairly, we benchmarked it against three widely used CNN architectures. ResNet50V2 [33] represents modern residual networks that are commonly applied and easily fine-tuned for medical imaging tasks. ResNet50V2 is the updated version of the ResNet network family that performs better than ResNet50 and ResNet101 on the ImageNet dataset. DenseNet201 [21] serves as a high-capacity model that aggressively reuses features, often achieving strong accuracy at the cost of increased computational demand. InceptionV3 [34] captures multi-scale patterns efficiently through parallel convolutions, offering a balance between performance and computational efficiency. These baselines collectively span residual, densely connected and multi-scale design paradigms, enabling a comprehensive assessment of LXNet in terms of accuracy, generalization and efficiency under identical training conditions.

### ResNet50V2

ResNet50V2 is a 50-layer deep CNN that leverages residual learning to enable the training of very deep architectures without suffering from vanishing gradients. The key component is the residual block, which introduces a shortcut (identity) connection allowing the input of a block to bypass one or more convolutional layers and be directly added to the block’s output:


$$\begin{array}{c} y_{l}=F(x_{l},W_{l})+x_{l} \end{array}$$ (14)

where $x_{l}$ is the input, F(·) represents the residual mapping (convolution, batch normalization, and activation), $W_{l}$ are the weights, and $y_{l}$ is the output. ResNet50V2 differs from the original ResNet by using pre-activation, applying batch normalization and ReLU before convolution, which improves gradient flow and training stability. The network concludes with global average pooling and a softmax classifier, providing strong accuracy while maintaining efficient training.

### DenseNet201

DenseNet201 is a 201-layer densely connected CNN. Unlike traditional CNNs, each layer receives inputs from all preceding layers, promoting feature reuse and mitigating the vanishing gradient problem [35]. Formally, the input to the *l*^th^ layer is:


$$\begin{array}{c} x_{l}=H_{l}([x_{0},x_{1},\ldots ,x_{l-1}]) \end{array}$$ (15)

where $\left[x_{0},x_{1},\ldots ,x_{l-1}\right]$ denotes the concatenation of feature maps from all previous layers, and $H_{l}$ is a composite function of batch normalization, ReLU, and convolution. This dense connectivity reduces the number of parameters, improves information flow, and enables accurate and efficient learning for complex image classification tasks.

### InceptionV3

InceptionV3 is designed to efficiently capture spatial features at multiple scales using parallel convolutions of different kernel sizes within the same module. The core *Inception module* applies 1 × 1, 3 × 3, and 5 × 5 convolutions in parallel, then concatenates their outputs:


$$\begin{array}{c} y=\text{Concat}({\text{conv}}_{1\times 1}(x),\;{\text{conv}}_{3\times 3}(x),\;{\text{conv}}_{5\times 5}(x)) \end{array}$$ (16)

where *y* is the output of the module, ${\text{conv}}_{1\times 1}(x)$, ${\text{conv}}_{3\times 3}(x)$, and ${\text{conv}}_{5\times 5}(x)$ denote the outputs of the respective convolution branches, and Concat(·) concatenates them along the channel dimension. This multi-scale feature extraction enables the network to learn rich representations while keeping computational costs manageable.

### Model training and optimal hyperparameters selection

LXNet was trained entirely from scratch using random weight initialization (Glorot Uniform), enabling the model to learn task-specific representations directly from the CXR images. In contrast, all baseline models (DenseNet201, ResNet50V2, and InceptionNet V3) were initialized with ImageNet pre-trained weights and fine-tuned using a transfer learning strategy, in which the convolutional backbones were retained and the original classification heads were replaced with task-specific heads corresponding to the target classes. To ensure a fair and consistent comparison, the baseline models were optimized within the same hyperparameter search space as LXNet, including optimizer type, learning rate, batch size, activation function, dropout rate and classification head configuration.

Table 1 summarizes the optimization spaces explored to identify the best-performing configurations for LXNet and the baseline models. For LXNet, systematic ablation and hyperparameter optimization experiments were conducted on the hold-out dataset, in which individual architectural components and training parameters, such as classifier head design, optimizer selection, learning rate, activation function, batch size and number of epochs, were independently varied while keeping other factors fixed. This ablation-driven optimization process enabled the identification of the most effective architectural and training configurations, ensuring that the final LXNet model achieved strong generalization performance while remaining computationally efficient. Subsequently, the best-performing configurations of all models were compared and the corresponding quantitative results are reported in the Results section.

**Table 1:** Optimization and Hyperparameter Search Space for LXNet and Baseline Models.

Optimization Space	LXNet	Baseline Models
Weight initialization	Random (Glorot Uniform)	Pre-trained (ImageNet)
Optimizer	SGD, RMSprop, Adamax, Adam, Nadam	
Learning rate	0.001, 0.0001, 0.0003, 0.0005	
Batch size	16, 32, 48, 64	
Max epochs	40	
Loss function	Categorical Cross-Entropy	
Activation function	LeakyReLU, ELU, Swish, ReLU	
Dropout rate	Spatial: 0.1; Dense: 0.3, 0.5	
Classification head	Flatten, GlobalMaxPooling2D, Dual Pooling, GlobalAveragePooling2D	

### 5-fold cross-validation

To ensure robust and unbiased performance evaluation, a 5-fold cross-validation strategy was employed using the cross-validation dataset, in which the images were partitioned into five mutually exclusive folds. Each fold was used once as a validation set, while the remaining four folds served as the training set. After training, performance metrics, including accuracy and macro F1-score, were computed for each fold and recorded. The fold-wise results were then aggregated and analyzed to compare the generalization performance of different models statistically.

The choice of a 5-fold configuration represents a practical balance between computational efficiency and statistical reliability. Using fewer folds (e.g., 3) could lead to higher variance in the results, while using more folds (e.g., 10) would significantly increase computational cost without a proportional gain in performance stability. The 5-fold approach thus provides a widely accepted trade-off suitable for deep learning tasks involving large image datasets. A stratified cross-validation strategy was employed to maintain proportional representation of all nine classes within each fold. This is particularly important for medical image datasets, which often exhibit varying class frequencies. Stratification ensured that the model was exposed to a representative sample of each class during both training and validation, preventing bias toward more prevalent disease categories and improving the reliability of performance estimates.

## Experiment results

### Ablation study and hyperparameter optimization results

A series of ablation experiments was performed to evaluate the effect of key hyperparameters on LXNet’s performance. Each parameter was varied independently, while the others were kept at their baseline settings. Model performance was assessed using validation and test accuracy and the summarized outcomes are presented in Tables 2–7.

**Table 2:** The impact of different classification head architectures on LXNet accuracy.

Head	Test ACC	Val ACC	Findings
Flatten	0.9778	0.9811	Lower test accuracy compared to pooling methods
GlobalMaxPooling2D	0.9833	0.9867	Slight improvement in both test and validation accuracy
Dual Pooling	0.9556	0.9611	Worst performance among tested heads
GlobalAveragePooling2D	0.9944	0.9922	Highest test and validation accuracy

**Table 3:** The effect of optimizers on accuracy for training the LXNet.

Optimizer	Test ACC	Val ACC	Findings
SGD	0.8989	0.8822	Poor test and validation accuracy, slow convergence
RMSprop	0.9878	0.9889	High test and validation accuracy, stable training
Adamax	0.9911	0.9911	Slightly higher test and validation accuracy than RMSprop
Nadam	0.9944	0.9922	Highest test accuracy, stable convergence
Adam	0.9944	0.9922	High accuracy, similar performance to Nadam

**Table 4:** The effect of learning rates on accuracy.

Learning rate	Test ACC	Val ACC	Findings
0.0001	0.9656	0.9829	Low test accuracy, slightly higher validation accuracy indicates underfitting
0.0003	0.9844	0.9829	Balanced training with high test and validation accuracy
0.0005	0.9911	0.9810	High test accuracy but slightly lower validation accuracy
0.001	0.9944	0.9525	Highest test accuracy but unstable validation performance

**Table 5:** Impact of the activation function on accuracy.

Activation function	Test ACC	Val ACC	Findings
LeakyReLU	0.9822	0.9733	Lower accuracy than Swish and ReLU
Swish	0.9878	0.9900	Highest test and validation accuracy
ReLU	0.9844	0.9811	Slightly lower than Swish
ELU	0.9678	0.9611	Lowest performance among tested activations

**Table 6:** The influence of batch size on accuracy.

Batch size	Test ACC	Val ACC	Findings
16	0.9878	0.9922	Good performance, slightly lower test accuracy than batch 48
32	0.9878	0.9900	Similar performance to batch 16
48	0.9911	0.9900	Highest test accuracy
64	0.9889	0.9833	Slight drop in both test and validation accuracy

**Table 7:** Impact of training epochs on convergence and accuracy.

Epoch	Test ACC	Val ACC	Findings
10	0.8811	0.8511	Underfitting due to insufficient training
20	0.9689	0.9578	Improved accuracy, better convergence
30	0.9878	0.9911	High accuracy, nearing convergence
40	0.9922	0.9967	Highest test and validation accuracy

### Classifier head

Different classifier head configurations were examined to identify the most effective strategy for aggregating spatial features before the final dense layer. As shown in Table 2, Flatten and DualPooling resulted in lower accuracy, while GlobalMaxPooling2D provided modest improvement. The highest performance was achieved with GlobalAveragePooling2D, achieving test accuracy of 0.9944 and validation accuracy of 0.9922. This confirms that global average pooling retains the most discriminative spatial information and is optimal for LXNet’s classifier head.

### Optimizer

The optimizer significantly influences convergence speed and training stability. LXNet was evaluated using SGD, RMSprop, Adamax, Nadam and Adam. As summarized in Table 3, traditional SGD underperformed (test accuracy < 0.90), while adaptive optimizers achieved superior results. Both Nadam and Adam achieved the highest test accuracy (0.9944), with Nadam selected for its smoother convergence and more stable validation performance.

### Learning rate

The learning rate determines the balance between training stability and convergence efficiency. As shown in Table 4, a small rate (0.0001) led to underfitting, while larger rates (0.0005−0.001) improved initial learning but caused instability. A learning rate of 0.0003 provided the best trade-off between convergence speed and final accuracy and was thus adopted as the optimal setting.

### Activation function

Activation functions play a vital role in learning complex, non-linear patterns. Table 5 compares the performance of ReLU, LeakyReLU, ELU, and Swish. The results indicate that Swish consistently outperformed the others, achieving the highest test (0.9878) and validation (0.9900) accuracies. The smooth and non-monotonic nature of Swish enables better feature representation, making it the preferred activation for LXNet.

### Batch size

Batch size influences both computational efficiency and model generalization. As shown in Table 6, smaller batches (16 and 32) yielded stable but slightly lower accuracy, while larger batches (64) reduced generalization. A batch size of 48 achieved the best performance, with a test accuracy of 0.9911 and strong validation stability, providing the most balanced configuration.

### Number of epochs

The number of epochs controls the extent of model convergence. As reported in Table 7, test accuracy increased steadily with training duration from 0.88 at 10 epochs to nearly 0.99 at 30 epochs. Optimal convergence was observed at 40 epochs, achieving a test accuracy of 0.9922 and validation accuracy of 0.9967. Beyond 40 epochs, improvements were marginal, indicating adequate saturation. Overall, the ablation findings establish the following optimal configuration for LXNet: GlobalAveragePooling2D classifier head, Nadam optimizer, learning rate of 0.0003, Swish activation, batch size of 48, and 40 training epochs. This combination yielded superior accuracy, robust convergence and efficient computational performance.

Based on the ablation studies presented in Tables 2–7, the optimal hyperparameter configuration for LXNet was determined. Table 8 summarizes the optimal hyperparameters identified through the ablation studies. The final configuration for LXNet includes the GlobalAveragePooling2D classifier head (test accuracy = 0.9944, validation accuracy = 0.9922), Nadam optimizer (selected for its stable convergence and high accuracy), learning rate of 0.0003 (balancing performance and stability), Swish activation function (outperforming ReLU, ELU and LeakyReLU), batch size of 48 and 40 training epochs (ensuring convergence with negligible gains beyond this point). This combination achieved the most balanced trade-off between accuracy, training stability and computational efficiency and was adopted as the final configuration for all subsequent experiments and model comparisons.

**Table 8:** Optimal hyperparameters for LXNet identified via ablation studies.

Hyperparameter	Best Option
Classifier Head	GlobalAveragePooling2D
Optimizer	Nadam
Learning Rate	0.0003
Activation Function	Swish
Batch Size	48
Epochs	40

### Comparison with baseline models under hold-out evaluation

Tables 9–12 shows the comparative performance of LXNet against the baseline models, along with the class-wise performances. The micro, macro, and weighted average performances of the models were included in the tables. Micro, macro, and weighted-average accuracies differ in both their calculations and interpretations. Micro-average accuracy is calculated by aggregating correct predictions over all samples, so each instance contributes equally and majority classes dominate the result. Macro-average accuracy is computed by first calculating accuracy for each class separately and then taking their unweighted mean, giving equal importance to all classes. Weighted-average accuracy is obtained by averaging class-wise accuracies weighted by the number of samples in each class. For imbalanced medical datasets, macro-average accuracy is more reliable for fair class-wise evaluation, while weighted-average accuracy reflects overall practical performance. Since we used a balanced dataset for model evaluation, we mainly reported accuracy using the micro average in this paper.

**Table 9:** Class-wise performance of LXNet on the hold-out test set.

Class	Accuracy	Precision	Recall	F1-score	Support
*Normal*	0.987	0.941	0.950	0.945	100
*Pneumonia*	0.987	0.949	0.930	0.939	100
*Higher Density*	1.000	1.000	1.000	1.000	100
*Lower Density*	0.997	0.980	0.990	0.985	100
*Obstructive Pulmonary Diseases*	0.996	0.980	0.980	0.980	100
*Degenerative Infectious Diseases*	0.999	1.000	0.990	0.995	100
*Encapsulated Lesions*	0.999	0.990	1.000	0.995	100
*Mediastinal Changes*	1.000	1.000	1.000	1.000	100
*Chest Changes*	1.000	1.000	1.000	1.000	100
**Micro Accuracy**				**0.982**	900
**Macro Avg**	0.996	0.982	0.982	0.982	900
**Weighted Avg**	0.996	0.982	0.982	0.982	900

**Table 10:** Class-wise performance of ResNet50V2 on the hold-out test set.

Class	Accuracy	Precision	Recall	F1-score	Support
*Normal*	0.978	0.857	0.960	0.906	100
*Pneumonia*	0.981	0.988	0.840	0.907	100
Higher density	1.000	1.000	1.000	1.000	100
*Lower Density*	0.999	0.990	1.000	0.995	100
*Obstructive Pulmonary Diseases*	0.998	0.990	0.990	0.990	100
*Degenerative Infectious Diseases*	0.997	0.980	0.990	0.985	100
*Encapsulated Lesions*	0.998	0.990	0.990	0.990	100
*Mediastinal Changes*	0.999	0.990	1.000	0.995	100
*Chest Changes*	1.000	1.000	1.000	1.000	100
**Micro Accuracy**				**0.974**	900
**Macro Avg**	0.994	0.976	0.974	0.975	900
**Weighted Avg**	0.994	0.976	0.974	0.975	900

**Table 11:** Class-wise performance of DenseNet201 on the hold-out test set.

Class	Accuracy	Precision	Recall	F1-score	Support
*Normal*	0.964	0.900	0.900	0.900	100
*Pneumonia*	0.971	0.911	0.820	0.863	100
*Higher Density*	0.989	0.990	0.970	0.980	100
*Lower Density*	0.996	0.900	1.000	0.947	100
*Obstructive Pulmonary Diseases*	0.982	0.948	0.920	0.934	100
*Degenerative Infectious Diseases*	0.994	0.925	0.990	0.956	100
*Encapsulated Lesions*	1.000	0.990	1.000	0.995	100
*Mediastinal Changes*	1.000	1.000	1.000	1.000	100
*Chest Changes*	1.000	1.000	1.000	1.000	100
**Micro Accuracy**				**0.956**	900
**Macro Avg**	0.989	0.952	0.956	0.953	900
**Weighted Avg**	0.989	0.952	0.956	0.953	900

**Table 12:** Class-wise performance of InceptionNet V3 on the hold-out test set.

Class	Accuracy	Precision	Recall	F1-score	Support
*Normal*	0.958	0.870	0.870	0.870	100
*Pneumonia*	0.947	0.851	0.690	0.762	100
*Higher density*	0.984	0.969	0.950	0.959	100
*Lower density*	0.994	0.891	0.990	0.938	100
*Obstructive Pulmonary Diseases*	0.980	0.899	0.950	0.924	100
*Degenerative Infectious Diseases*	0.972	0.902	0.920	0.911	100
*Encapsulated Lesions*	1.000	0.962	1.000	0.981	100
*Mediastinal Changes*	1.000	0.980	1.000	0.990	100
*Chest Changes*	0.999	0.980	1.000	0.990	100
**Micro Accuracy**				**0.930**	900
**Macro Avg**	0.981	0.923	0.930	0.925	900
**Weighted Avg**	0.981	0.923	0.930	0.925	900

**Fig 5:**
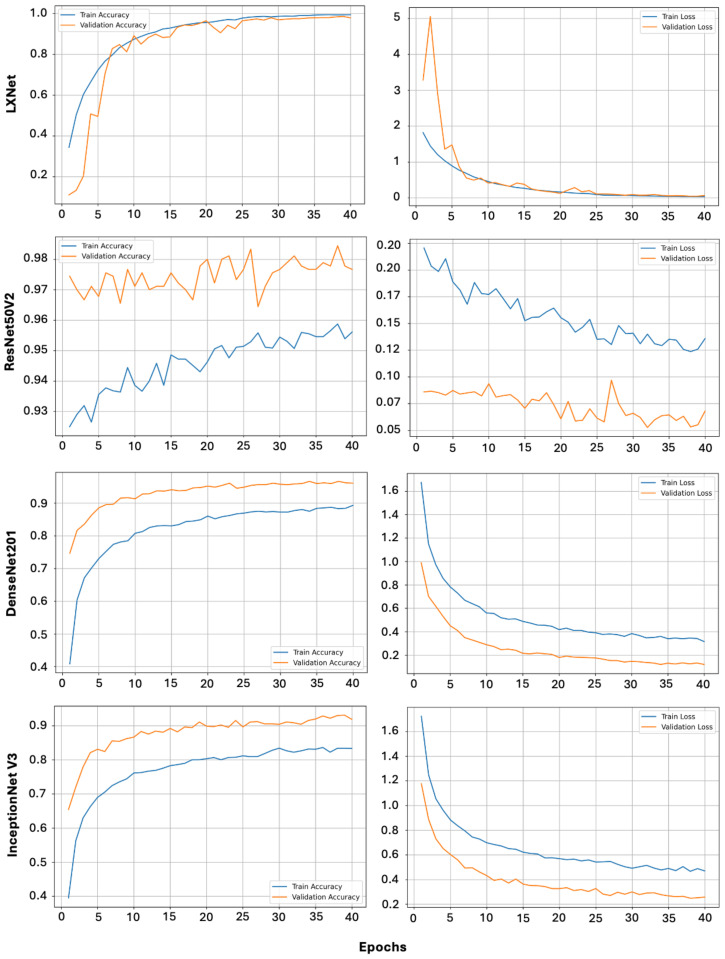
Training and validation curves for LXNet under the final configuration.

Among the baselines, InceptionNetV3 demonstrated the lowest micro accuracy of 0.93, indicating limited generalization capability. DenseNet201 achieved moderately higher, more consistent performance (0.956 across all metrics) but required longer training due to its dense feature propagation. ResNet50V2 performed slightly better, reaching 0.974 with improved convergence stability. All the models are trained under identical settings to ensure fair comparison. LXNet achieved the highest micro accuracy of 0.982. This consistent improvement of 1–9% over the pretrained baselines demonstrates the effectiveness of LXNet’s lightweight design in capturing discriminative features while maintaining computational efficiency. The results confirm that LXNet delivers robust, efficient performance suitable for practical applications where accuracy and speed are both critical. Fig 5 shows the training and validation accuracy and loss curves for the LXNet.

### Class-wise performance of the models on the hold-out test

Table 9 shows the per-class accuracy, precision, recall and F1-score of LXNet across the nine classes. The model demonstrates outstanding performance, achieving perfect precision (1.00) in *Higher Density*, *Degenerative Infection Diseases*, *Mediastinal Changes*, and *Chest Changes*. *Obstructive Pulmonary Diseases* also reached an accuracy of 0.99, with a minor reduction in precision (0.98), indicating minimal misclassifications. The *Normal* class attained slightly lower values (accuracy = 0.98, precision = 0.94, recall = 0.95, F1 = 0.94), reflecting minor confusion with other classes that exhibit subtle radiographic patterns. *Pneumonia* had an accuracy of 0.98, a recall of 0.93, yielding an F1-score of 0.93, suggesting that a few positive cases were misclassified, likely due to overlapping radiographic features with other pulmonary conditions. *Degenerative Infectious Diseases* achieved an F1-score of 0.99, with perfect precision but slightly lower recall (0.99), indicating a small number of false-positive predictions. Overall, LXNet achieved macro and weighted-average accuracies of 0.99, highlighting consistent and balanced performance across all classes. These results confirm that LXNet can reliably discriminate subtle and challenging thoracic patterns, demonstrating robustness and minimal misclassification even in the most difficult categories.

The comparison of class-wise performances from Table 9, 10, 11 and 12 shows that LXNet consistently achieves the highest or near-highest scores across all classes. For the *Normal* class, LXNet attains an F1-score of 0.945, outperforming ResNet50V2 (0.906), DenseNet201 (0.900), and InceptionNet V3 (0.870). A similar trend is observed for *Pneumonia*, where LXNet achieves an F1-score of 0.939, compared to 0.907 (ResNet50V2), 0.863 (DenseNet201), and 0.762 (InceptionNet V3), indicating a clear margin of improvement. For structurally distinct classes such as *Higher density*, *Mediastinal changes*, and *Chest changes*, both LXNet and ResNet50V2 achieve perfect F1-scores of 1.000, while DenseNet201 and InceptionNetV3 show slightly lower or comparable results. In intermediate-complexity classes, including *Lower density* and *Obstructive pulmonary diseases*, LXNet maintains F1-scores above 0.98, marginally exceeding ResNet50V2 and clearly outperforming DenseNet201 and InceptionNetV3. Overall, LXNet demonstrates the most balanced and robust class-wise performance, particularly for difficult and relevant categories, as reflected in its highest micro accuracy (0.982) among all evaluated models.

**Fig 6:**
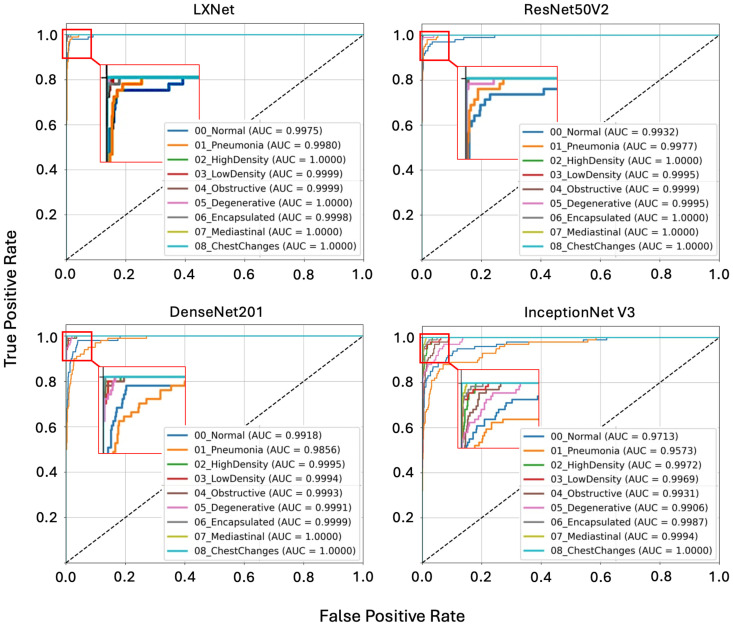
ROC curve of the Models.

**Fig 7:**
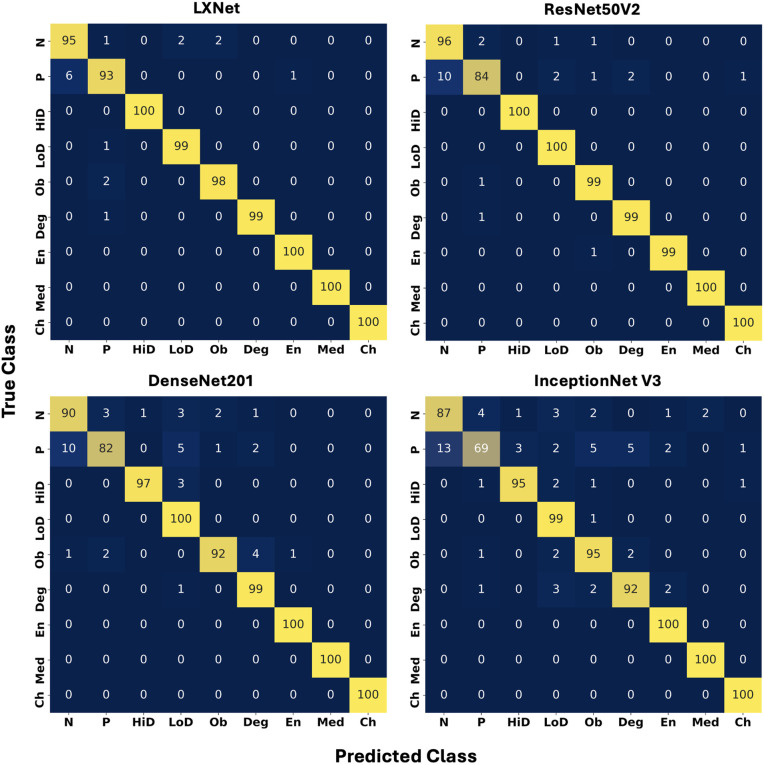
Confusion matrix of the Models for test data(N = *Normal*, P = *Pneumonia*, HiD = *Higher Density*, LoD = *Lower Density*, Ob = *Obstructive Pulmonary*, Deg = *Degenerative Infectious Diseases*, En = *Encapsulated Lesions*, Med = *Mediastinal Changes*, Ch = *Chest Changes*).

Fig 6 and 7 show the Receiver operating characteristic (ROC) curves and confusion matrices of the models for the test set, providing a detailed view of class-wise prediction performance. LXNet demonstrated the most balanced performance across all nine classes. For the Normal class, LXNet correctly classified 95 samples, with only minor confusion into *Pneumonia* (1) and *Lower Density* (2), whereas ResNet50V2, DenseNet201, and InceptionNet-V3 misclassified 4, 10 and 13 cases, respectively, indicating progressively weaker discrimination of healthy tissue. A similar pattern is observed for *Pneumonia*, where LXNet achieved 93 correct predictions with limited confusion, compared to higher misclassification in ResNet50V2 (16 cases) and substantially greater dispersion in DenseNet201 and InceptionNet V3, the latter misclassifying 31 samples across multiple categories. For *Obstructive Pulmonary Diseases* and *Degenerative Infectious Diseases*, LXNet maintained strong robustness with 98 and 99 correct predictions, respectively. At the same time, DenseNet201 and InceptionNet V3 showed higher cross-class confusion, reflecting reduced stability in differentiating overlapping pathological patterns.

While ResNet50V2 achieved comparable performance to LXNet, particularly for structurally distinct classes, it displayed higher confusion in *Normal–Pneumonia* and *Pneumonia–Degenerative* pairs compared to LXNet. DenseNet201 and InceptionNet V3 showed the highest inter-class confusion, especially among *Normal, Pneumonia, Obstructive and Degenerative* classes, indicating reduced robustness in ambiguous cases. Consistent with the confusion matrix analysis, LXNet achieved superior class-wise consistency without sacrificing performance across any category, as further supported by its uniformly high AUC values across all classes (over 0.99). Collectively, these results demonstrate that LXNet not only matches or exceeds baseline models in overall accuracy but also provides more reliable and interpretable class-level predictions, particularly in challenging scenarios.

### 5-Fold cross-validation analysis

We performed 5-fold cross-validation experiments three times independently, using different randomly generated seeds (42, 123 and 999). This provides a more rigorous assessment of robustness and reproducibility. As shown in Table 13, LXNet achieved consistently strong performance across all folds, with fold-wise accuracies ranging from 0.952 to 0.973. The overall average accuracy was 0.961 ± 0.007, demonstrating both high predictive performance and low variability across folds and seeds. The relatively small standard deviations across folds further indicate that LXNet is stable with respect to random initialization and data shuffling, confirming its reliable generalization without signs of overfitting. In comparison, the baseline pretrained models exhibited either slightly lower mean performance or greater instability. ResNet50V2 achieved a comparable average accuracy of 0.960 ± 0.007; however, its fold-wise fluctuations suggest marginally less consistent behavior across different splits and seeds. DenseNet201 obtained a lower average accuracy of 0.903 ± 0.004, while InceptionNetV3 achieved 0.889 ± 0.004, both indicating substantially weaker performance. Moreover, DenseNet201 showed relatively larger per-fold standard deviations (up to 0.034), highlighting increased sensitivity to data partitioning and random initialization. Overall, the repeated cross-validation protocol (multiple folds combined with multiple seeds) strengthens the reliability of the findings. The results confirm that LXNet maintains competitive accuracy with improved stability across diverse training conditions, supporting its robustness and suitability for practical deployment.

**Table 13:** 5-Fold cross-validation accuracy of models over three random seeds (Avg = average; Std = standard deviation).

Fold	LXNet	ResNet50V2	DenseNet201	InceptionNetV3
Fold 1	0.971 ± 0.009	0.947 ± 0.012	0.909 ± 0.019	0.890 ± 0.002
Fold 2	0.952 ± 0.014	0.967 ± 0.004	0.899 ± 0.031	0.893 ± 0.006
Fold 3	0.962 ± 0.013	0.963 ± 0.002	0.896 ± 0.034	0.882 ± 0.004
Fold 4	0.973 ± 0.010	0.964 ± 0.002	0.907 ± 0.025	0.892 ± 0.014
Fold 5	0.960 ± 0.019	0.957 ± 0.009	0.905 ± 0.031	0.885 ± 0.005
Avg ± Std	**0.961 ± 0.007**	**0.960 ± 0.007**	**0.903 ± 0.004**	**0.889 ± 0.004**

Further, a paired *t*-test was conducted on the repeated 5-fold cross-validation results (5 folds × 3 seeds). The analysis showed that LXNet significantly outperformed DenseNet201 and InceptionNetV3 (*p* < 0.05), while no statistically significant difference was observed between LXNet and ResNet50V2 (*p* > 0.05), indicating comparable performance between these two models.

### Robustness analysis

**Fig 8:**
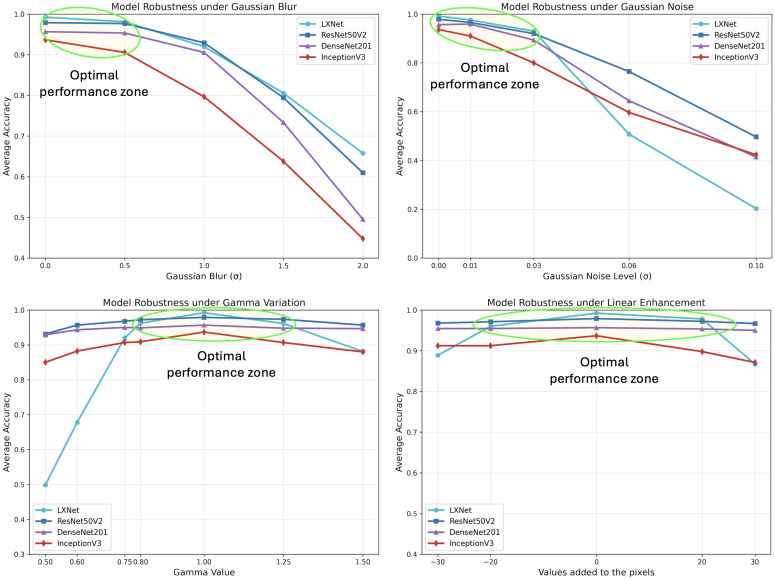
Performance of the models for digitally simulated test sets.

To evaluate model robustness under common image degradation conditions in CXRs, we performed a systematic analysis considering Gaussian blur, Gaussian noise, Gamma correction (non-linear transformation) and linear intensity adjustments. Gaussian blur images were generated with standard deviations from 0 to 2.0, Gaussian noise with standard deviations from 0 to 0.10, Gamma values ranged from 0.50 to 1.50 and linear intensity shifts were simulated by adding or subtracting fixed values to pixel intensities. The analysis revealed that LXNet maintained high performance under moderate Gaussian blur, while ResNet50V2, DenseNet201 and InceptionV3 showed sharper degradation. Under Gaussian noise, ResNet50V2 was most stable, whereas LXNet was more sensitive at higher noise levels. LXNet showed reduced performance at very low Gamma values but recovered near a Gamma of 1.0, while ResNet50V2 and DenseNet201 remained stable across Gamma variations. All models tolerated linear intensity shifts reasonably well, with LXNet and ResNet50V2 performing slightly better. Within moderate degradation ranges, LXNet’s performance remained stable, highlighted as the optimal performance zone in Fig 8.

We further evaluated LXNet on an external dataset collected from multiple sources and laboratories to assess domain shift, as shown in Table 14. The model achieved a micro-averaged accuracy of 0.706, performing well on normal, pneumonia, high-density, mediastinal and chest classes, but showing reduced accuracy for less common or complex classes, such as obstructive pulmonary diseases and degenerative infectious diseases. These results indicate that while LXNet is robust under moderate degradation and generalizes well to common patterns, its performance decreases for rarer or more complex pathological variations.

**Table 14:** Class-wise performance of LXNet on external dataset.

Class	Accuracy	Precision	Recall	F1-score	Support
*Normal* [36]	0.900	1.000	0.900	0.947	20
*Pneumonia* [37]	0.950	0.613	0.950	0.745	20
*High Density* [38]	0.950	0.760	0.950	0.844	20
*Low Density* [36]	0.650	0.650	0.650	0.650	20
*Obstructive Pulmonary Diseases* [36,39]	0.300	0.400	0.300	**0.343**	20
*Degenerative Infectious Diseases* [40,36]	0.200	0.267	0.200	**0.229**	20
*Encapsulated Lesions* [36]	0.400	0.571	0.400	**0.471**	20
*Mediastinal Changes* [36]	1.000	0.952	1.000	0.976	20
*Chest Changes* [38]	1.000	0.952	1.000	0.976	20
**Micro Accuracy**				**0.706**	180
**Macro Avg**	0.706	0.685	0.706	0.687	180
**Weighted Avg**	0.706	0.685	0.706	0.687	180

### Statistical significance

To evaluate whether the observed performance differences between LXNet and the baseline models (ResNet50V2, DenseNet201 and InceptionNetV3) were statistically meaningful, a non-parametric analysis was conducted using the results of repeated 5-fold cross-validation across three random seeds (42, 123 and 999). Parametric tests such as the paired *t*-test assume independence and normality of samples; however, these assumptions are violated in *k*-fold cross-validation due to overlapping training sets and shared data distributions across folds. Therefore, we employed the Wilcoxon signed-rank test, which is suitable for paired, small-sample comparisons and does not rely on distributional assumptions. For each baseline model, fold-wise accuracies were paired with those of LXNet and the accuracy difference for each fold was computed as


$$\begin{array}{c} d_{i}=A_{i}^{\text{LXNet}}-A_{i}^{\text{baseline}},\;i=1,\ldots ,5, \end{array}$$ (17)

where $A_{i}$ denotes the classification accuracy on the *i*-th fold. Zero differences were excluded and the remaining absolute differences $\left|d_{i}\right|$ were ranked in ascending order. Each rank was assigned the sign of the corresponding difference. The Wilcoxon signed-rank test statistic was then computed as:


$$\begin{array}{c} W=\sum \text{sign}(d_{i})\;\text{rank}(|d_{i}|), \end{array}$$ (18)

and evaluated using a two-sided hypothesis test with the null hypothesis


$$\begin{array}{c} H_{0}:\text{median}(d_{i})=0. \end{array}$$ (19)

The Wilcoxon signed-rank test revealed clear performance patterns between LXNet and the baseline models. Compared with ResNet50V2, LXNet achieved a negligible mean accuracy gain of +0.001; this difference was not statistically significant (*p* = 0.3125), indicating that LXNet and ResNet50V2 perform comparably. In contrast, LXNet significantly outperformed DenseNet201, with a mean accuracy gain of +0.058 (*p* = 0.03125) and InceptionNetV3, with a larger mean accuracy gain of +0.072 (*p* = 0.03125). These results confirm that LXNet consistently provides a meaningful advantage over DenseNet201 and InceptionNetV3, while achieving parity with ResNet50V2. To further strengthen robustness, performance was averaged across the three random seeds in addition to the 5 folds, yielding an overall mean ± standard deviation of 0.961 ± 0.007 for LXNet, compared to 0.960 ± 0.007 for ResNet50V2, 0.903 ± 0.004 for DenseNet201 and 0.889 ± 0.004 for InceptionV3. These results highlight LXNet’s low variability across both folds and seeds. Moreover, LXNet maintained consistently high class-wise accuracy across all nine classes, whereas baseline models showed greater inter-class confusion. Collectively, these findings demonstrate that LXNet provides more stable, reproducible and superior generalization performance under repeated cross-validation conditions.

### Comparison with existing methods

Table 15 shows the comparison of LXNet with recent lung disease classification models. The method proposed by Aldamani et al. [31] used EfficientNetB2 and achieved 98.50% accuracy. The Versatile ML Architecture System of Rodriguez-Carpio [41] achieved 97.20% accuracy and Gutta’s RespiraAI [42] achieved 95.00% accuracy. LXNet outperformed these recently methods, reaching 99.62% (macro average accuracy in hold out test), while maintaining consistently high per-class F1-scores (0.97–1.00) and statistically significant improvements over baselines (*p* < 0.000001, large Cohen’s *d*). These results highlight the effectiveness of LXNet’s lightweight design, optimized hyperparameters and feature recalibration, demonstrating both high predictive accuracy and robust generalization suitable for practical use.

**Table 15:** Comparison of LXNet with recent CNN-based lung disease classification methods.

Reference	Model	Test Accuracy
Aldamani et al. (2024) [31]	EfficientNetB2	98.50%
Rodríguez-Carpio et al. (2024) [41]	Versatile ML Architecture	97.20%
Gutta (2025) [42]	RespiraAI	95.00%
This work (2026)	LXNet	99.62%

### Interpretability analysis

**Fig 9:**
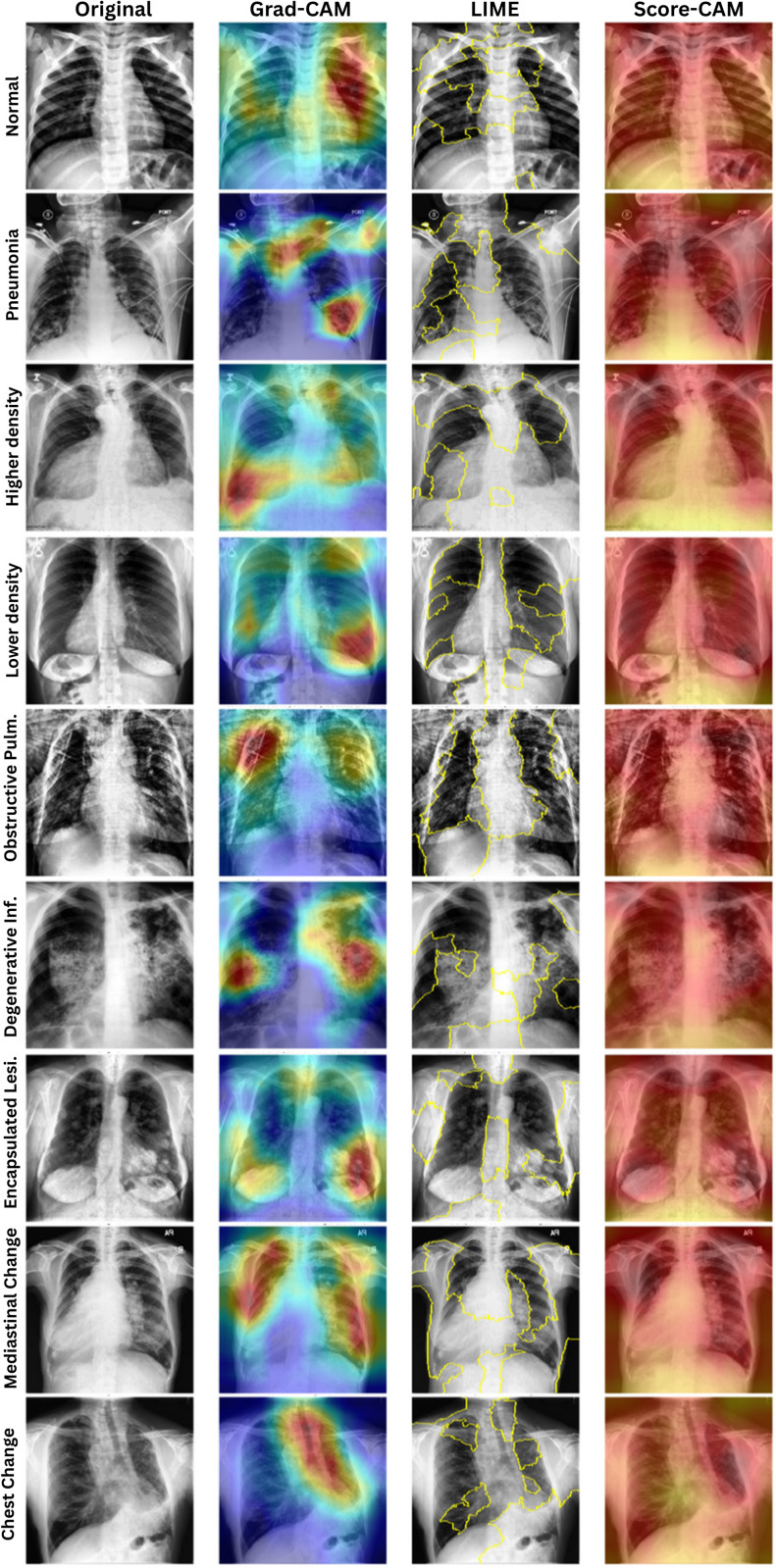
Visual explanations of LXNet predictions using Grad-CAM, Score-CAM, and LIME.

To enhance trust and support real-world adoption, we evaluated the interpretability of LXNet using three complementary XAI techniques: Grad-CAM [16], Score-CAM [43] and LIME [44] across all nine lung disease categories. Representative examples are shown in Fig 9.

Grad-CAM highlights class-discriminative regions by weighting convolutional feature maps with the gradient of the predicted class score. In the heatmaps (second column of Fig 9, Grad-CAM consistently emphasized relevant thoracic regions, such as diffuse opacities in *Pneumonia*, localized *Encapsulated Lesions* and altered density patterns in *Obstructive Pulmonary Disease*. Score-CAM offers a gradient-free alternative that weights activation maps based on their contribution to the classification score. Compared to Grad-CAM, Score-CAM maps were smoother and less noisy, enabling clearer localization of affected regions and reducing sensitivity to gradient saturation, particularly useful for subtle radiographic abnormalities. LIME approximates local decision boundaries by perturbing superpixels. The third column in Fig 9 shows the super-pixels most influential to each prediction. While less spatially precise than CAM-based methods, LIME provided complementary insights by highlighting meaningful subregions, such as lobar clusters or mediastinal areas, that contributed to the model’s confidence.

To further illustrate disease-specific feature learning, we analyzed how LXNet’s attention maps align with established radiological patterns. For *Pneumonia*, Grad-CAM and Score-CAM highlighted patchy opacities, particularly in the lower lung zones, consistent with infectious infiltrates. In *Encapsulated Lesions*, activations were highly localized around well-defined nodular regions, indicating sensitivity to sharply bounded abnormalities. For *Obstructive Pulmonary Disease*, the model emphasized radiolucent regions and altered lung textures over broader areas, reflecting hyperinflation-related changes. In *Normal* CXRs, attention was diffusely distributed across clear lung fields, suggesting reliance on the absence of pathology. LIME-based superpixel analysis further supported these findings by identifying coherent anatomical regions, such as lobar and perihilar areas, contributing to predictions. Overall, these results indicate that LXNet captures disease-specific imaging features rather than spurious correlations.

Across all classes, LXNet consistently focused on meaningful pulmonary regions rather than spurious background artifacts, demonstrating reliable attention behavior. By integrating multiple XAI techniques, the analysis confirms that the model’s predictions are interpretable and grounded in relevant features. This not only validates the robustness of LXNet across diverse thoracic conditions but also supports its key contribution as an accurate and transparent AI tool for assisting radiologists in complex CXR interpretation. Future work may incorporate complementary explainability approaches, such as SHapley Additive exPlanations (SHAP) and Prediction Difference Analysis (PDA), to provide both global and local interpretability and further enhance reliability.

### Time complexity analysis

Table 16 presents a detailed comparison of training times for LXNet and three baseline pretrained CNN models, including per-epoch and total training durations, along with their micro average test accuracy on the hold out set. LXNet exhibits remarkable computational efficiency, requiring only 32 seconds for the first epoch and an average of 7.7 seconds per subsequent epoch, resulting in a total training time of 308 seconds. Despite its lightweight design, LXNet achieved the highest test accuracy of 0.982 (micro average test accuracy). In comparison, ResNet50V2 and InceptionNet V3 required 721 and 668 seconds in total, achieving 0.974 and 0.930 micro average test accuracy, respectively, while DenseNet201 incurred the longest training time of 1320 seconds with a slightly lower accuracy of 0.956. These results demonstrate that LXNet delivers superior predictive performance while maintaining fast training, making it highly suitable for resource-constrained environments and rapid deployment.

**Table 16:** Comparative training duration and accuracy of LXNet versus baseline CNNs (Avg = average; Sec = seconds; Acc = accuracy).

Model Name	First Epoch (sec)	Avg Epoch (sec)	Total Time (sec)	Micro Avg Test Acc
InceptionV3	44	16.7	668	0.930
ResNet50V2	33	18.03	721	0.974
DenseNet201	116	33	1,320	0.956
**LXNet**	**32**	**7.7**	**308**	**0.982**

## Discussion

In this study, we have demonstrated the accuracy and validated the lightweight, interpretable properties of LXNet through experiments. The proposed LXNet achieved state-of-the-art performance in multi-class lung disease classification of CXR images. As shown in Table 15, LXNet attained an overall accuracy of 99.62%, surpassing previous models. LXNet was trained and evaluated alongside established pretrained architectures such as DenseNet201, ResNet50V2, and In- ceptionNet V3 under identical conditions to provide a fair and comprehensive benchmark. To ensure robust model design, comprehensive ablation studies were conducted to identify the optimal hyperparameters for LXNet, including the classifier head (*GlobalAveragePooling2D*), optimizer (*Nadam*), learning rate (0.0003), activation function (*Swish*), batch size (48) and number of epochs (40). This systematic evaluation confirmed that the chosen configuration balances predictive accuracy, training stability and computational efficiency. Additionally, 5-fold stratified cross-validation experiments were performed to validate the model’s robustness across diverse data splits. It should be noted that the reported performance metrics were obtained under different evaluation protocols. Specifically, the 96.1% accuracy corresponds to the five-fold cross-validation setting, whereas the 99.62% macro average accuracy was achieved using the hold-out test set. Furthermore, Wilcoxon signed-rank tests confirmed that the observed improvements over pretrained CNN baselines were statistically and practically significant.

Class-wise performance and confusion matrix analyses further highlight LXNet’s reliability, with perfect classification across 7 of 9 classes and only minor misclassifications in the Pneumonia and Normal categories. Macro- and weighted-average F1-scores of 0.99 indicate consistent performance across all pulmonary patterns. Furthermore, efficiency comparisons with pretrained networks demonstrate that LXNet achieves high accuracy (macro average accuracy of 99.62 and micro average accuracy of 98.20) with substantially lower computational cost, requiring only 308 seconds for total training compared to 1,320 seconds for DenseNet201 and 721 seconds for ResNet50V2. Interpretability analyses using Grad-CAM, Score-CAM and LIME reveal that LXNet’s predictions are based on useful thoracic regions, including patchy opacities in Pneumonia, localized encapsulated lesions, and diffuse patterns in Obstructive Pulmonary Diseases. By triangulating insights from multiple XAI techniques, we ensured that the model’s decision-making is transparent and interpretable. Overall, the proposed method achieves the key claims of being lightweight, interpretable and highly accurate. Its optimized architecture and hyperparameters provide computational efficiency, cross-validation and statistical analyses confirm robustness, and explainable AI techniques ensure meaningful and interpretable reasoning. Collectively, these attributes establish LXNet as a promising solution for rapid, reliable and interpretable multi-class lung disease classification from CXR images.

Despite LXNet’s strong performance, several limitations remain. A key limitation of this study is the lack of patient-level metadata in the utilized CXR dataset, including the number of unique patients and demographic information such as age, sex, ethnicity and clinical background. As a result, the representativeness and potential demographic biases of the dataset cannot be fully assessed, limiting conclusions about model generalizability across diverse populations. Furthermore, due to the absence of patient identifiers, image-level data splitting was performed, and patient-level duplication or data leakage cannot be entirely ruled out.

Secondly, the model was trained and primarily evaluated on a single publicly available dataset. Although we further validated the trained model on an external dataset containing images collected from different laboratories, larger-scale evaluation is necessary to fully establish its generalizability. In real-world settings, CXR images may vary significantly across imaging centers due to differences in scanner types, acquisition protocols, image resolution and patient populations, which may affect model performance. The external validation conducted in this study was relatively small in scale and mainly assessed model performance under domain shift conditions. In addition, robustness experiments using noisy, blurred and Gamma-shifted images provided an estimate of the operational limits of the proposed model under controlled degradations. However, real-world variability is often more complex and heterogeneous. Therefore, future work should include validation on multiple large-scale, multi-center datasets and more diverse imaging conditions to comprehensively confirm the robustness and generalizability of the proposed method. It should also be noted that, due to the unavailability of patient identifiers and associated patient-level metadata (e.g., age, sex and clinical background), image-level splitting was adopted during data partitioning. Consequently, patient-level duplication or data leakage cannot be completely excluded, which may result in an optimistic estimation of model performance. Therefore, the robustness and real-world clinical applicability of the proposed model should be interpreted cautiously. In addition, the absence of clinical metadata limits the assessment of model generalizability across diverse patient populations and imaging conditions. Future studies should employ strict patient-level separation and incorporate comprehensive clinical information to further validate the robustness and clinical reliability of the proposed approach.

Third, the current framework focuses solely on disease classification and does not include severity grading because the dataset lacks severity-level annotations, thereby restricting its utility for more specific diagnosis. Future work will aim to incorporate severity estimation frameworks to enhance the model’s reliability. Fourth, although LXNet is a computationally efficient model, further optimization, such as quantization, pruning or hardware-aware compression, is required for real-time deployment on mobile or embedded devices.

Finally, this study has not yet undergone prospective clinical validation by radiologists, which is a necessary step before real-world deployment. In addition, the observed performance drop on external datasets highlights limitations in generalizability, particularly across certain disease categories. Although the proposed system was not deployed in a clinical environment, its readiness was evaluated against multiple practical criteria, including diagnostic accuracy, robustness, interpretability and computational efficiency, all of which are critical for real-world adoption. Future work will focus on large-scale external validation and expert-led evaluation to further assess usability, reliability and diagnostic impact.

Therefore, the future work should address these key limitations: (i) cross-dataset validation to ensure robustness across varied acquisition protocols and image quality; (ii) severity assessment to extend the model’s capabilities from classification to grading of disease severity, enhancing practical applicability; (iii) integration with multi-modal data by combining CXR analysis with patient metadata (e.g., age, symptoms, laboratory tests) for richer diagnostic insights; (iv) real-time deployment through further optimization for mobile and embedded systems; and (v) prospective validation with radiologists to evaluate how explainable AI outputs support decision-making and trust calibration. Addressing these directions could transform LXNet from a high-performing research prototype into a deployable diagnostic tool.

## Conclusion

This study introduced LXNet, a lightweight and interpretable CNN designed for multi-class lung disease classification from CXR images. Through comprehensive evaluation and comparison with state-of-the-art pretrained models, LXNet achieved high accuracy (macro-average accuracy of 99.62% on the hold-out test and 96.1% under 5-fold cross-validation) and computational efficiency. The integration of XAI techniques further demonstrated the model’s capacity to make meaningful and transparent predictions. These findings suggest that LXNet is a promising and resource-efficient network for pulmonary disease screening and diagnostic support. However, the absence of patient-level metadata limits the assessment of dataset representativeness and potential bias and should be addressed in future studies.
